# Diet of Three Cryptobenthic Clingfish Species and the Factors Influencing It

**DOI:** 10.3390/ani14192835

**Published:** 2024-10-01

**Authors:** Domen Trkov, Danijel Ivajnšič, Marcelo Kovačić, Lovrenc Lipej

**Affiliations:** 1 Marine Biology Station Piran, National Institute of Biology, Fornače 41, 6330 Piran, Slovenia; lovrenc.lipej@nib.si; 2 Jožef Stefan Institute and Jožef Stefan International Postgraduate School, Jamova Cesta 39, 1000 Ljubljana, Slovenia; 3 Faculty of Natural Sciences and Mathematics, University of Maribor, Koroška Cesta 160, 2000 Maribor, Slovenia; dani.ivajnsic@um.si; 4 Faculty of Arts, University of Maribor, Koroška Cesta 160, 2000 Maribor, Slovenia; 5 Natural History Museum Rijeka, Lorenzov Prolaz 1, 51000 Rijeka, Croatia; marcelo@prirodoslovni.com

**Keywords:** non-destructive method, fish, diet habits, faecal pellets, *Lepadogaster lepadogaster*, *Lepadogaster candolii*, *Apletodon incognitus*, crustaceans, northern Adriatic Sea

## Abstract

**Simple Summary:**

Clingfish are small fish species that spend most of their time hiding in various shelters on the seabed. Due to this way of life, their ecology is little known, although they are important for the ecosystem. The aim of the research was to investigate the diet of three clingfish species (*Lepadogaster lepadogaster*, *L. candolii*, and *Apletodon incognitus*) using a method based on the analysis of prey from their faeces. The results show that crustaceans are the most important prey for all three species, although the composition of the diet also depends on various factors, such as the size of the fish and the prey, the behaviour of the fish, the home range of the fish, and the availability of food. These results provide us with important information about the participation of clingfish in the food web and deepen our knowledge of the fish’s diet and the factors that influence it. The results also show that the occurrence of predatory fish depends on the presence of their prey.

**Abstract:**

Cryptobenthic fish are small benthic fish species that normally live in various hiding places. Due to their large numbers, they are very important for energy transfer to higher trophic levels. However, due to their small size and hidden lifestyle, knowledge about them and their ecology, including their diet, is still limited. Using a non-destructive method based on faecal pellets, we investigated the diet of three clingfish species, *Lepadogaster lepadogaster*, *L. candolii*, and *Apletodon incognitus*, in the shallow northern Adriatic Sea. To better understand the results, we studied the fauna of potential prey in the habitats of the fish studied and also took fish specimens to observe their behaviour in the laboratory. The three species feed predominantly on crustaceans, particularly amphipods, copepods, and decapods. The proportion of the different taxa in the diet depends on the species of clingfish, the size of the specimens, and the size of the prey. In addition, the behaviour of the fish, the home range of the specimens, and the availability of food played an important role. The presence of certain crustacean groups in the environment also determines the occurrence of clingfish of different species and sizes.

## 1. Introduction

A very important part of the fish communities in coastal waters is cryptobenthic fish fauna. A fish species or a life history stage of a fish species is cryptobenthic if individuals exclusively or predominantly spend their lifetime in cryptobenthic microhabitats, that is, in the restricted living spaces underneath the bottom surface of the substrate or biocover, with a physical barrier to open spaces [1]. Due to this lifestyle, they are rarely observed by divers and are usually not detected during conventional ichthyofauna surveys [2]. Therefore, knowledge on the ecology of cryptobenthic fishes remains very incomplete and their importance has been mostly overlooked in the past. Smith-Vaniz, Jelks, and Rocha [3] reported that about 64% of the fish fauna sampled with rotenone in their Caribbean research was not detected by visual observation. In the Mediterranean, Kovačić et al. [4], using three different methods, recorded 42 fish species in total. The two visual census methods recoded 31 species, while the anaesthetic method found 18 species, with an overlap of 7 species that were ambivalent in their occurrence. This is probably one of the reasons why cryptobenthic fish species were considered rare in the past [5], while more recent studies have shown that they are quite common and abundant in the Mediterranean [6] (and references therein [7]). Studies on cryptobenthic fishes from tropical reefs have even shown that they have the potential to influence the ecosystem due to their high abundance and therefore represent an important part of biodiversity in coastal areas that can significantly influence ecosystem functions [8]. While many authors report on the importance (e.g., energy transfer) of cryptobenthic fish species in tropical seas [8,9,10], there is still a lack of knowledge on cryptobenthic fish species from the Mediterranean and their ecology [7,11,12].

One of the least known taxa of cryptobenthic fish species in the Mediterranean Sea is clingfishes (family Gobiesocidae) [7,13]. Clingfishes have a flattened, scaleless body and are mainly smaller than 10 cm [14,15]. They are characterised by a suction disc consisting of pelvic fins, a reduced swim bladder [14,16], and the absence of a stomach [17]. Three species have been recorded in Slovenian waters so far, namely *Lepadogaster lepadogaster* (Bonnaterre, 1788), *L. candolii* (Risso, 1810), and *Apletodon incognitus* Hofrichter and Patzner, 1997 [18,19,20]. *L. lepadogaster* and *L. candolii* are the most widespread European clingfish species, distributed throughout the Mediterranean and also along the eastern Atlantic coast from England to northwest Africa, the Canary Islands, and Madeira [2]. *A. incognitus* has been only recently described [21] and little is known about its ecology. The species is known to occur in the northern Mediterranean Sea, including the Adriatic Sea, and in the eastern Atlantic Ocean near the Azores [21,22]. Although all three species differ in their choice of habitats and especially depth distribution, there are also small overlaps in their habitat use that could be influenced by food supply [20]. Understanding feeding relationships is very important as it could reveal different strategies to reduce competition, such as resource partitioning [23]. In addition, knowledge of foraging habits provides information about ecological processes at the individual, population, and community levels [24]. Feeding and foraging habits also determine the position (trophic level) of animals in food webs and define their ecological role [25]. However, most studies on the fish diet are based on the examination of stomach contents. The main disadvantage of this method is that the studied animals must be killed and dissected, so for many species studied, large numbers of animals must be sacrificed [24,26], which can lead to significant changes in the population structure of fish species in some areas [27,28]. The potential negative effects of over-extraction on *L. lepadogaster* populations have already been highlighted by King [29]. At the same time, such methods are prohibited in marine protected areas (MPAs), and it is unlikely to obtain a permit to study legally protected species by using lethal methods [20]. Since more than half of the natural Slovenian coastline is protected by a 200 m wide marine belt and belongs to MPAs [30], the use of non-destructive sampling methods is essential. With this in mind, we applied a non-destructive method developed for the study of small benthic species, which has been shown to be very effective for *L. lepadogaster* [31].

The aims of the study are: (1) to apply a non-destructive method to three clingfish species, *L. lepadogaster*, *L. candolii*, and *A. incognitus*, and evaluate it; (2) to study the diets of these species and their position within the food webs; and (3) to determine factors influencing their diet composition. Within each species, we studied the diet of the specimens of the different sexes, size classes, and from different bottom depths. In the case of *L. lepadogaster*, seasonal differences in diet were also studied.

## 2. Materials and Methods

### 2.1. Study Area

The study was conducted in the Slovenian part of the Gulf of Trieste, the northernmost part of both the Adriatic Sea and the Mediterranean Sea. The Gulf of Trieste is a shallow gulf (20–25 m) with an area of about 500 km^2^ [32]. The coastline of the Slovenian part of the Gulf of Trieste is about 46 km long [33]. The coastal bottom consists of boulder fields and gravel banks that extend to a depth of about 10 m, from where it is replaced by sediment bottom [20]. Soft sedimentary bottom of fluvial origin can also be found in the shallow waters of the bays [32]. Seagrass meadows (mostly *Cymodocea nodosa*) are found on the shallow sediment bottom at depths between 1 and 11 m [34]. Most of the flysch cliffs have retained their natural state, while the coastal plains are heavily exposed to anthropogenic influences [33].

The salinity in the Gulf of Trieste is typically marine but is influenced by freshwater inflows, ranging from 33 to 38.5‰. The water temperature normally fluctuates from 8 °C in winter to 24 °C in summer [32]. The average tidal range is high compared to the rest of the Adriatic Sea, with the low water level up to 80 cm below the mean sea level [35,36,37,38]. The Isonzo River is the most important freshwater source, which has a significant influence on seasonal plankton dynamics (autotrophic plankton and zooplankton). In addition, the development of the autotrophic plankton biomass determines the patterns of the consumer community [39].

### 2.2. Field Work

#### 2.2.1. Fish Sampling

**Figure 1:**
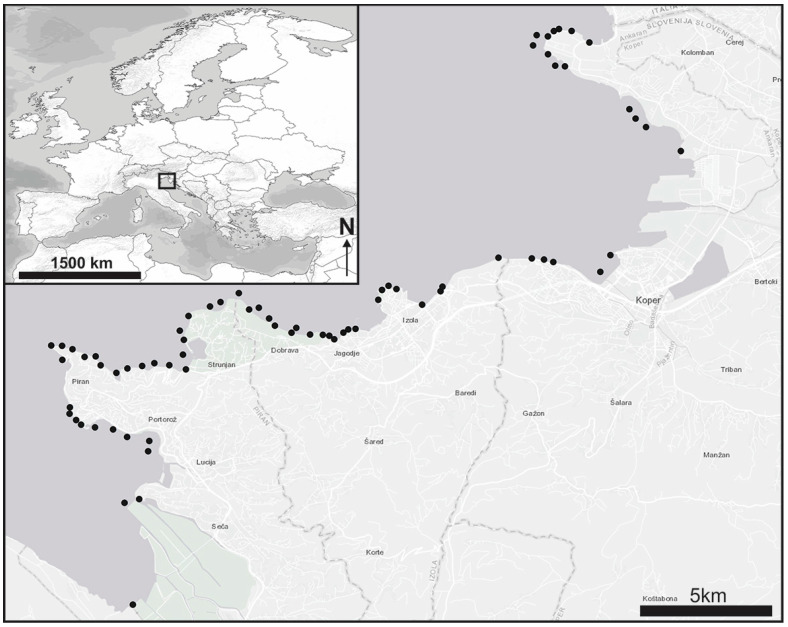
Sampling sites (black dots) along the Slovenian coast where clingfish were searched for.

The sampling of cryptobenthic fish was carried out from January 2017 to March 2019 at various locations in the Slovenian part of the Gulf of Trieste. All sampling was carried out in coastal waters. The specimens were collected by snorkelling and by SCUBA diving in the infralittoral zone. In addition, some specimens were also collected in the mediolittoral and tidal pools at low tide. The main habitat types sampled were boulder fields, gravel banks, seagrass meadows with *Cymodocea nodosa* (Ucria) Ascherson, 1870 and *Posidonia oceanica* (Linnaeus) Delile, 1813, as well as sandy-muddy bottoms [20]. A total of 206 sampling sessions of approximately 1 h each were conducted at 72 randomly selected sites (Figure 1). 

The random search for fishes was performed in various hiding places, e.g., under stones, rocks, shells or in natural cavities such as caves, holes, crevices, etc. To facilitate the collection of the fish, the narcotic Quinaldine (Sigma-Aldrich St. Louis, MO, USA) was used. Quinaldine was diluted at 1:15 with alcohol [40,41]. The anaesthetic was sprayed into the hiding place using a laboratory wash bottle. The anaesthetised fish were then caught with a hand net. Specimens of *L. lepadogaster* were also collected by lifting stones and capturing specimens with a hand net (d = 40 cm). Specimens attached to the underside of the stone were then dropped directly into the net. Specimens of *A. incognitus* inhabiting oyster shells were captured by placing a plastic bag over the oyster and then chasing the fish into the bag. The captured specimens were kept in a 100 mL plastic chamber with a lid, which had small holes for the inflow of oxygenated water which were small enough to prevent faecal pellets from passing through.

At each collecting site, the following data were recorded: date, location, macrohabitat (e.g., seagrass meadow, rocky area, sediment bottom), microhabitat (e.g., rock pile, noble pen shell with dead oysters), hiding place (e.g., under a rock, in an oyster shell), and bottom depth. For sampling in the mediolittoral, the depth was calculated using the depth of the sampling site and the zero point from the tidal amplitude calendar [36,37,38].

#### 2.2.2. Invertebrate Sampling

To investigate the structure of the fauna in the clingfish habitat, samples of benthic invertebrates were taken in May 2018 (warm season) and January 2019 (cold season). We sampled the shelters most frequently used by the clingfish species. Under stones inhabited by *L. lepadogaster*, 10 sample squares (25 × 25 cm, 625 cm^2^) were sampled in the cold season and 10 sample squares in the warm season, while under stones inhabited by *L. candolii*, 10 sample squares were collected only in the warm season, as they were most frequently caught at this time. In the shelters with clingfishes, macroinvertebrates were collected with the substrate (e.g., pebbles, sand), using a hand net (20 × 20 cm, mesh size 500 µm). The square size of 25 × 25 cm was chosen because a frame of 20 × 20 cm (400 cm^2^) is considered the minimum area for sampling Mediterranean infralittoral communities [42]. Substrate and macroinvertebrates were stored in plastic bags and brought to the Marine Biology Station Piran, National Institute of Biology (MBS-NIB). Since *A. incognitus* is associated with *Pinna nobilis* Linnaeus, 1758 (Trkov et al. [18], an endangered species whose populations are declining [43], no faunal samples were taken in those shelters.

### 2.3. Laboratory Work

#### 2.3.1. Fish

After sampling, the fish were brought to the MBS-NIB as quickly as possible i.e., in less than one hour, where they were photographed with the Olympus TG-4, measured to the nearest 0.01 mm with a calliper (total length) and weighed to the nearest 0.01 g with a Sartorius TE 1502S balance (wet weight). Particular attention was paid to the condition of the clingfish, which were always kept moist during the measurements. The fish were identified at the species level using scientific literature with species diagnoses, descriptions, and identification keys [2,14,21,44,45]. The sex of specimens was determined based on the length and size of the urogenital papillae, which are larger and more elongated in males than in females [11,15]. For the size-based analyses, the specimens of each species were, preliminarily tested for size differences using Levene’s test for equality of variances and the corresponding ANOVA analysis with Tukey contrasts post hoc test in the R statistical environment and then divided into different size classes. The specimens of *L. lepadogaster* and *L. candolii* were divided by Hofrichter [11] into five size classes, while those of *A. incognitus* were divided into four size classes. For ease of interpretation, the size classes correspond to the age-size classes proposed by Hofrichter ([11]; Table 1). However, the age estimate based on size could contain a considerable error, as the age ranges of the fish cohorts usually overlap, so the age estimate is only a rough approximation, therefore we have not used it in our analyses, except in Table 1. Due to the small sample size in some size classes, size classes in this research were merged as follows: *L. lepadogaster*: ≤3, 4, and 5; *L. candolii:* 1, 2, 3, and ≥4; *A. incognitus:* ≤2, 3, and ≥4. 

#### 2.3.2. Invertebrate Fauna

The substrate collected in the fish shelters was examined for invertebrates as available food for clingfish. The invertebrates were identified using the scientific literature with species diagnoses, descriptions, and identification keys [46,47,48,49] and counted. The main prey taxa were also measured and weighed (Olympus SZx16 stereomicroscope with an Olympus DP74 camera; Sartorius CP 225D balance; wet weight from 70% ethanol) to obtain length–weight correlation curves for application on ingested prey.

#### 2.3.3. Fish Diet

The non-destructive method based on faecal pellets [31] was used to determine fish diet. After measuring and weighing, each freshly caught clingfish was placed in an 11 × 13 × 14 cm chamber with filtered seawater (125 µm) and an aerator above the bottom to prevent the defragmentation of the faecal pellets. Freshly caught clingfish were left in the chambers for 24 h. Every three to four hours (during the day), the animals were checked to see if they had produced faecal pellets. The pellets were carefully removed from the bottom of the chambers using a modified pipette and fixed in 70% EtOH. Pellets consist of a peritrophic membrane and the inner part with undigested prey pieces. In some cases, the pellets were broken, and the contents of the entire chamber were filtered through a 125 µm mesh plankton net and checked. After defecation, the specimens were released unharmed at the site where they had been collected. After each use, the chambers were disinfected with 70% EtOH to prevent the transmission of diseases and parasites. Based on a small sample of freshly captured *A. incognitus* examined using a non-destructive method, we also include eight specimens from the MBS-NIB collection (seven captured in 2007 and one in 2019) whose gut contents were examined by dissection.

The contents of the faecal pellets and gut were analysed under an Olympus stereomicroscope and photographed with a camera. Some faecal pellets were also weighed using a Sartorius CP 225D balance (wet weight of 70% EtOH). Prey were identified to the lowest taxa level possible to be identified and then counted. A large proportion of the digested prey was whole or almost whole, while digested prey, which was broken down into smaller pieces, was recognised by typical body parts such as the carapace. The prey that was most digested, such as polychaetes and amphipods, were identified by their jaws [31]. 

The wet weight of the most important prey (e.g., decapods) was calculated using correlation curves between the length and weight of the available food collected in the sampling area (from samples preserved in 70% EtOH) (see Section 2.3.2). Undigested prey and prey that only occasionally appeared in the faeces or prey whose weight could not be calculated from the correlation curves, were weighed directly from the faecal pellets, or the average weight (of the animals collected in the sampling area) was used.

#### 2.3.4. Observations in Aquaria

Three specimens of each species, i.e., *L. lepadogaster*, *L. candolii*, and *A. incognitus* were housed in three aquaria at MBS-NIB, where they were observed for better understanding of their feeding habits and behavioural patterns. The aquaria measured 150 × 50 × 50 cm and were set up to mimic the natural habitat of the clingfish. For *L. lepadogaster* and *L. candolii*, the aquaria were set up to mimic boulder fields with pebbles and larger stones, while *A. incognitus* was housed in an aquarium that mimicked a sediment bottom with a *Pinna nobilis* shell and oysters on top. The behaviour of each fish was observed for three days, 10 min in the morning and 10 min in the afternoon, for a total of 60 min per species. Occasionally, some observations were also made by night out of the scheduled observations.

#### 2.3.5. Data Analysis

Three different quantitative methods were used to capture the complexity of the studied fish diet: frequency of occurrence (F%; [50]), numerical abundance (N%; [51]), and gravimetric composition (B%; [51]). In addition, the modified index of relative importance (IRI) [52,53] was calculated using the following formula: IRI = F% (N% + B%), where F% is the frequency of occurrence, N% is the numerical abundance, and B% is the gravimetric composition. To facilitate comparisons between different foods, Cortés [54] recommended expressing the IRI as a percentage. The IRI% for a given food category f is IRI % = (IRI/∑IRI) × 100. 

The dietary diversity of prey was expressed by the index of trophic diversity (ITD), which is a modified Shannon–Wiener diversity index (H′; [55]): ITD = 1 − H′. ITD values range from 0 (no diversity) to 1 (full diversity). The ITD was calculated for all three species and for different size groups. 

To investigate the trophic level of the species, the TROPH value was calculated for all three species and for different size groups. The trophic level was calculated as follows [25,56,57]: TROPHi = 1 + ∑DCij × TROPHj, where DCij represents the proportion of prey j in the diet of consumer species i; TROPHj represents the proportion of prey j in the trophic level. The program TrophLab (a stand-alone Microsoft Access routine for estimating trophic levels; June 2000 version) was downloaded from www.fishbase.org (accessed on 5 March 2019) [56] and used to calculate the TROPH index of the species studied. For means, standard deviation (SD) was used to calculate. 

A nonparametric permutative multivariate analysis of variance (PERMANOVA) based on the abundance data of the recorded prey taxa in the faecal pellets and the Bray–Curtis dissimilarity measure matrix was used to evaluate the diet differences in the numerical abundance of prey among studied species and to check for the diet differences by sex, size, bottom depth, and seasons for each species. In addition, a linear regression test (*t*-test) was used to test the linear dependence between the size of the prey and the size of the fish.

To compare diet among species, we used the Bray–Curtis dissimilarity index, which provides information on ecological or dietary niche overlap [58,59]: BCij = 1 − (2Cij/(Si + Sj)), where Cij is the sum of the lower values only for the prey taxa shared by both clingfishes. Si and Sj are the total number of prey taxa counted in the diet of each species. Values range from 0 (no overlap) to 1 (complete overlap). To show the importance of each prey taxa at different depths, the SIMPER function was used [60]: d[ijk] = abs(x[ij] − x[ik])/sum(x[ij] + x[ik]), where x is the abundance of prey taxa i in sampling units j and k. The total index is the sum of the individual contributions across all S prey taxa d[jk] = sum(i = 1…S) d[ijk]. The SIMPER function performs pairwise comparisons of groups of sampling units and determines the average contributions of the individual prey taxa to the average overall dissimilarity according to Bray–Curtis (https://www.rdocumentation.org/packages/vegan/versions/2.6-2/topics/simper; accessed on 14 May 2020). When using the summary function (ordered = TRUE), the results include the cumulative contribution and are ordered by the contribution of prey taxa to the potential difference in prey composition and abundance.

PERMANOVA and all other tests were calculated using the R statistical environment [61] and the Vegan package (version 2.6-2) [62], if not noted differently at the particular test above. 

For the invertebrate fauna collected in the habitat of *L. lepadogaster* and *L. candolii*, the relative frequency of occurrence (F%) and the relative abundance of individuals of the different taxa (N%) per species habitat were calculated.

## 3. Results

### 3.1. Diet Study of Three Clingfish Species

A total of 363 specimens of *L. lepadogaster*, *L. candolii*, and *A. incognitus* were included in the study (Table S1). The non-destructive method was performed on 356 specimens (Table 2), all of which survived until their release into the wild. Faecal pellets were obtained from 96.9% of the specimens. Due to the small sample size of *A. incognitus*, we included eight specimens from the MBS-NIB collection whose gut contents were analysed.

#### 3.1.1. *Lepadogaster lepadogaster*

A total of 194 specimens were collected. Their total length ranged from 28.06 mm to 78.02 mm (on average, 57.60 ± 8.54 mm), while their weight ranged from 0.23 g to 5.85 g (2.17 ± 0.96 g). Among the 182 sexually identifiable specimens, 78 were females and 104 were males (sex ratio 1:1.3). The measurements were performed on 80 faecal pellets. The average length of the pellets was 4.64 mm, the average width was 2.63 mm, and the average weight was 16.64 mg.

**Figure 2:**
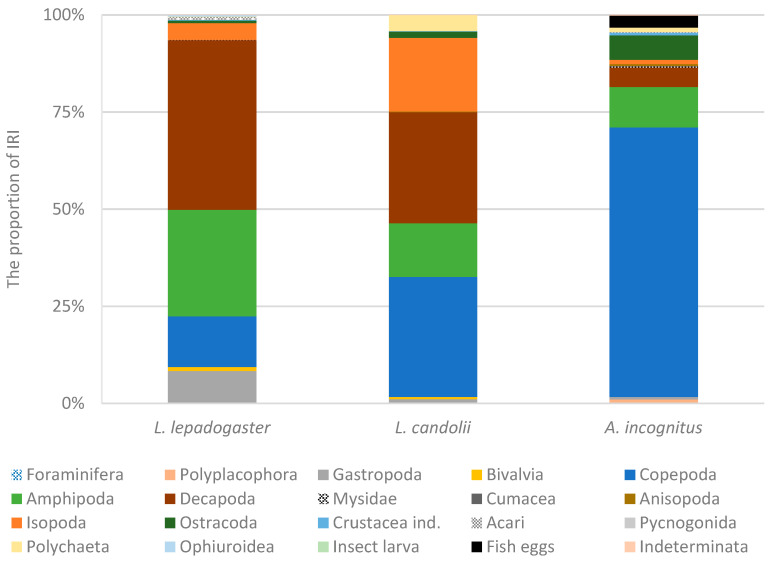
Index of the relative importance (%) of different prey groups for three clingfish species.

A total of 47 taxa, which were divided into 18 main taxonomic groups, were identified as prey. In addition to prey, the faecal pellets often contained various sediment particles such as sand grains, invertebrate shell fragments, and algal particles. Sediment particles could account for up to 16.5% of the weight of the faecal pellets. Copepods (N% = 30.1), mostly represented by harpacticoids, were by far the most numerous prey group, followed by amphipods (N% = 20.3) and decapods (N% = 12.6). The latter two prey categories were also the most abundant prey groups in terms of the frequency of occurrence. Amphipods were ingested by 67.6% of the specimens, while decapods were preyed on by 56.4% of the fish. The most important prey group in terms of biomass were decapods (B% = 60.9), followed by amphipods (B% = 18.3), which together accounted for the majority (79.2%) of the biomass. Decapods were the most important prey group in terms of relative importance (IRI% = 43.6; Figure 2) and were mainly represented by the crab *Pisidia* sp. (82.7% of the numerical abundance of decapods). According to the diagnostic characteristics, the crabs of the genus *Pisidia* mainly belong to *P. bluteli* (Risso, 1816) and less frequently to *P. longimana* (Risso, 1816). The results showed that the size of *Pisidia* sp. was not linearly related to the size of the fish (*t*-test for linear regression; *p* = 0.3216, α = 0.05). Alternative prey groups were amphipods (IRI% = 27.5), such as species of the family Gammaridae (97.4% of amphipods), and copepods (IRI% = 13.0), mostly harpacticoids. Gastropods, such as juveniles of *Bittium reticulatum*, *Alvania* sp., and *Rissoa* sp., accounted for 8.3% of the IRI. Based on the diet composition, we calculated the TROPHs index, which was 3.18 ± 0.44, and the ITD index, which was 0.83. 

The quantitative composition of the diet of *L. lepadogaster* was not significantly different between sexes (PERMANOVA; *p* = 0.648, α = 0.05) and also no significant difference was found in diet composition between the different size classes (PERMANOVA; *p* = 0.135, α = 0.05). However, copepods dominated the diet of the size group ≤ 3 years and their abundance and frequency of occurrence decreased with the size of the group. This trend was also observed in Acari and ostracods. In contrast, the abundance and frequency of occurrence of decapods, isopods, and gastropods increased with the size of the group. The TROPHs index ranged from 3.18 ± 0.36 for the smallest size group and increased to 3.35 ± 0.52 for the largest size group, while the ITD ranged from 0.75 in size group ≤3 to 0.82 for size group 5 (Table 3).

The differences in the diet composition among individuals at the different bottom depths were statistically significant (PERMANOVA; *p* = 0.001, α = 0.05). The most significant prey group in shallow water was copepods, while in deeper water it was decapods and gastropods. In addition, the SIMPER function showed that decapods were the most important prey group at the maximum depth of *L. lepadogaster* occurrence and their importance decreased with decreasing bottom depth. It also showed that the importance of copepods decreased with increasing depth. In addition, the numerical abundance of prey showed that copepods and bivalves were more abundant in shallower waters. In contrast, the abundance and frequency of occurrence of decapods, ophiuroids, and gastropods increased with increasing depth.

The seasonal differences in the diet composition were statistically significant (PERMANOVA; *p* = 0.001, α = 0.05). The presence of amphipods in the diet was most significant in winter, copepods in spring, and decapods in summer and autumn. In winter, the most abundant groups were amphipods and gastropods, which were also the most abundant prey groups in the diet. In spring, copepods were by far the most abundant prey group, accounting for over 52% of the numerical abundance. In summer, the numerical abundance of copepods decreased, while the abundance of decapods increased and became the most abundant and frequent prey group in the diet. The abundance of isopods, acari, and amphipods also increased in summer. In the autumn, amphipods were the most abundant prey group, followed by decapods, whose abundance decreased slightly compared to summer. However, the frequency of decapods in the diet was highest in the autumn (79.3%). A comparison of the size of *Pisidia* sp. crabs consumed shows that fish consumed the largest specimens of *Pisidia* sp. in winter, while their size decreased over the seasons and was smallest in autumn. In addition, seasonal differences in the diet of males were separately tested and were statistically significant (PERMANOVA; *p* = 0.001, α = 0.05), with the presence of copepods in the diet being most intensive in spring.

#### 3.1.2. *Lepadogaster candolii*

A total of 120 specimens were included in the dietary study. The total length of the 113 specimens measured ranged from 13.97 mm to 86.94 mm (average 41.48 ± 16.00 mm), while their weight ranged from 0.03 g to 6.05 g (1.08 ± 1.10 g). Of the 57 sexually identifiable specimens, 17 were male and 40 were female (sex ratio 1:2.4). A total of 119 faecal pellets were measured. The average weight of the faecal pellets was 10.42 mg and they were on average 3.53 mm long and 2.10 mm wide.

A total of 42 different prey taxa were found and recognised in the diet. The prey was categorised into 18 major taxonomic groups. In addition to prey remains, the faecal pellets sometimes contained various sediment particles such as sand grains, old invertebrate shell fragments, and algal particles, which could account for up to 64.1% of the weight of the faecal pellets. The most frequently preyed groups were copepods (F% = 74.2), followed by decapods (F% = 60.0), isopods (F% = 59.7), and amphipods (F% = 56.7). Copepods were by far the most numerous prey (N% = 47.2), followed by isopods (N% = 11.3). In terms of biomass, decapods were the most important (B% = 46.3), followed by isopods (B% = 25.4) and amphipods (B% = 20.5). Copepods (IRI% = 30.9) and decapods (IRI% = 28.5) were the most important prey in terms of IRI. Copepods were almost exclusively represented by harpacticoids. In addition, seven parasitic copepods (family Caligidae) were found in the diet. Decapods were mainly represented by the crabs of the genus *Pisidia* (55.0% of all decapods) and *Athanas nitescens* (38.3% of all decapods). They belonged mainly to *P. bluteli* and less frequently to *P. longimana*. Other prey groups were isopods (IRI% = 18.9) and amphipods (IRI% = 13.8). Among the isopods, many parasitic larvae of Gnathiidae were found in the diet, and in one case, fish scales were also found in the faecal pellet (probably ingested together with the parasite). The TROPHs index was 3.12 ± 0.36 and the ITD was 0.73.

The quantitative composition of diets showed no statistical difference between the sexes (PERMANOVA; *p* = 0.308, α = 0.05). Copepods were the most significant and most abundant prey group for both sexes. However, copepods, gastropods, and bivalves were more abundant in the diet of males, while decapods, isopods, and polychaetes were more abundant in the diet of females. In females, decapods (82.7%) were the most frequent prey group, followed by copepods (71.2%), while in males, copepods and amphipods (both 62.5%) were the most frequent prey groups, followed by decapods (56.3%). The difference in diet composition between specimens from different size classes was statistically significant (PERMANOVA; *p* = 0.002, α = 0.05). The most important prey groups for smaller juvenile specimens were copepods and polychaetes, while adult specimens preferred decapods. Copepods were most abundant and frequent in the diet of smaller clingfishes, and their proportion decreased with increasing group size, while the abundance and frequency of occurrence of decapods and isopods increased with increasing group size. The ITD index ranged from 0.63 in size group 1 to 0.81 in size group ≥4, while the TROPHs index ranged from 3.03 ± 0.27 in size group 1 to 3.22 ± 0.40 in the largest size group ≥4.

The differences in the diet composition among individuals from the different bottom depths were statistically significant (PERMANOVA; *p* = 0.001, α = 0.05). The abundance and frequency of occurrence of copepods and bivalves in the diet increased with increasing bottom depth, while the number of gastropods and decapods decreased with increasing depth. In addition, the SIMPER function showed that decapods were the most important prey group in the 2 to 3 m depth range and their importance decreased with increasing depth. The importance of copepods did not change with increasing depth, as shown by the SIMPER function. Due to the uneven distribution of samples across all four seasons, seasonal differences in diet were not analysed for *L. candolii*.

#### 3.1.3. *Apletodon incognitus*

A total of 45 specimens were included in the dietary analysis, of which 37 specimens were examined using the faecal pellet method, while the gut contents were examined in eight specimens from the MBS-NIB collection. The total lengths of the 38 specimens measured ranged from 15.06 mm to 45.21 mm (average 32.38 ± 9.27 mm), while their weights ranged from 0.03 g to 1.29 g (0.48 ± 0.37 g). Of the 34 sexually identifiable specimens, 9 were female and 25 were male (sex ratio 1:2.8). A total of 41 faecal pellets were measured. The pellets weighed an average of 1.95 mg and were 2.34 mm long and 1.58 mm wide.

In the diet, 23 different taxa, which were classified into 18 main taxonomic groups, were recognised. In some cases, in addition to the prey remains, various sediment particles such as sand grains, invertebrate shell fragments, and algal particles accounted for up to 35.1% of the faecal pellet weight. Most of the prey in the diet of *A. incognitus* were copepods (N% = 69.0), which were also the most frequent prey (F% = 77.8), followed by ostracods (F% = 55.6) and amphipods (F% = 46.7). The most important prey groups in terms of biomass were decapods (B% = 22.3), amphipods (B% = 17.5), and copepods (B% = 14.6). The decapod crustaceans were mainly represented by various shrimp species (e.g., *A. nitescens*) and crabs of the genus *Pisidia*. By far the most important prey based on the IRI were copepods (IRI% = 69.4), mainly represented by harpacticoids, followed by amphipods (IRI% = 10.4), which were mainly represented by Gammaridea (88.0% of amphipods) and less by species of the family Caprellidae. Alternative prey groups were ostracods (IRI% = 6.2) and decapods (IRI% = 5.1). Based on the diet composition, a TROPHs index of 3.13 ± 0.26 and an ITD of 0.51 were calculated.

The quantitative composition of diet showed a statistical difference between males and females (PERMANOVA; *p* = 0.003, α = 0.05). However, it should be noted that the sample of females was very small. Copepods, fish eggs, and ostracods were most significant in the male diet. Fish eggs were only found in the diet of the males. In both species, however, copepods predominated in terms of number and the frequency of occurrence. In females, ostracods, copepods, and decapods were the most frequent prey groups.

The difference in diet composition between the different size groups was statistically significant (PERMANOVA; *p* = 0.009, α = 0.05). In all three size groups, copepods were the most significant and numerous prey group. The smallest specimens had the most diverse diet, with a higher abundance and frequency of occurrence of amphipods, decapods, anisopods, and isopods compared to the two larger groups. Only in the largest group, which consisted only of males, fish eggs were found. In addition, there was a significant difference in diet composition between nesting and non-nesting males (PERMANOVA; *p* = 0.022, α = 0.05), with fish eggs being the most significant prey of nesting males. The ITD and TROPHs indices were also calculated for each size group. The TROPHs index ranged from a minimum of 2.98 ± 0.36 in the largest size group ≥4 to 3.26 ± 0.44 in size group ≤2. The ITD index was lowest in size group 3 (0.31), while it was highest in size group ≤2 (0.78).

Based on the bottom depth range, specimens were divided into three depth groups (2–3 m, 3–4 m, > 4 m), but no statistical significance was found in diet composition between specimens in the different groups (PERMANOVA; *p* = 0.247, α = 0.05). Copepods were the most abundant and frequent prey group in all three depth classes. Due to the uneven distribution of samples across all four seasons, seasonal differences in diet were not analysed.

#### 3.1.4. Comparison between Species

Crustaceans were the most important prey group for all clingfish species. However, there were significant differences in the diet composition among species (PERMANOVA; *p* = 0.001, α = 0.05) that significantly differed (*p* < α; α = 0.05) in size. Copepods were the most significant prey group in the diet of *A. incognitus* and to a slightly lesser extent of *L. candolii*, for which amphipods, decapods, and isopods were the most significant prey group. For *L. lepadogaster*, amphipods and decapods were the most significant prey groups, which is also confirmed by the IRI (%), which is highest for these two prey groups. The Bray–Curtis dissimilarity index showed that the greatest overlap in diet was between *L. candolii* and *L. lepadogaster* and the least overlap was between *A. incognitus* and *L. lepadogaster* (Table 4), which is confirmed by the IRI (%) of the different prey groups for all three species. In contrast to the diet of *L. lepadogaster*, where decapods were mainly represented only by crabs of the genus *Pisidia*, the diet of *L. candolii* included a large number of *A. nitescens* (38.3% of decapods) in addition to *Pisidia* sp. (55.0% of decapods). This was also observed in the fauna samples under stones, where *A. nitescens* made up 7.8% of the decapods in the lower mediolittoral and 26.5% in the infralittoral, where most of the *L. candolii* were found. The TROPHs index per species was highest in *L. lepadogaster* (3.18 ± 0.44), followed by *A. incognitus* (3.13 ± 0.26) and *L. candolii* (3.12 ± 0.36), while the ITD index was highest in *L. lepadogaster* (0.83), followed by *L. candolii* (0.73) and was lowest in *A. incognitus* (0.51).

### 3.2. Fauna in the Clingfish Habitat

The sampling of prey groups under stones inhabited by *L. lepadogaster* showed that amphipods were one of the most numerous and frequent prey groups in the area, followed by decapods (Table 5). A total of 1364 specimens of different prey groups were found in 20 sampling quadrants (25 × 25 cm^2^) in the lower mediolittoral and upper infralittoral.

The sampling of prey groups under stones inhabited by *L. candolii* showed that amphipods, decapods, polychaets and ophiuroids were the most numerous and frequent prey groups in this area. A total of 913 specimens of different prey groups were found in 10 sampling quadrants (25 × 25 cm) in the 2–3 m depth range. Due to the method used to sample the fauna (mesh size of the net), no information could be provided on the abundance and occurrence of copepods and other meiofaunal organisms in the area.

### 3.3. Fish Observations in the Aquaria

The movements of *L. lepadogaster* were mainly restricted to the underside of the rock. Most of the time they wait in ambush until the prey comes close enough to grab it, or they approach the prey with small jumps, when necessary, but they were never observed leaving the shelter.

*L. candolii* proved to be much more mobile compared to *L. lepadogaster*. The specimens usually hid by clinging to the underside of a rock, but they were also observed moving around the shelter. *L. candolii* not only waited in ambush for prey but actively searched for it everywhere in the aquarium. Small specimens were observed to hide less, especially at night (observed outside the scheduled observation periods) when they came out of the shelters and picked up copepods from the aquarium walls.

The specimens of *A. incognitus* in the aquarium behaved similarly to *L. candolii*. Most of the time, they hid in oyster shells, but they were also observed to move around. The specimens of *A. incognitus* waited in ambush for prey. When they spotted a potential prey, they approached with small jumps and then snatched it, like *L. candolii*. It was also observed that the clingfish did not attack when the prey was not moving. This behaviour was observed when they were feeding on specimens of the shrimp *Hippolyte* sp.

## 4. Discussion

### 4.1. Feeding Habits of Three Clingfish Species

Since all fish survived the faecal collection method, it can be considered non-destructive and suitable for use in MPAs and for protected fish species, as previously stated by Trkov and Lipej [31]. The faecal pellet-based method proved to be very useful and efficient for use with clingfish, as they were obtained from 96.9% of all specimens examined. The specimens of all three species often produce more than one faecal pellet, indicating a high degree of prey filling given their very short digestive tract. This is consistent with Depczynski and Bellwood [10], who found that cryptobenthic fish feed continuously or on energy-rich food. A high proportion (93%) of *L. lepadogaster* specimens that had prey in their gut was also reported by King [29], confirming the observations of Hofrichter [11] that *L. lepadogaster* fed throughout the day. This is also confirmed by observations on specimens of *A. incognitus*, which showed that the animals produce an average of 3.3 faecal pellets per day (one faecal pellet every 7–8 h; D.T. personal observation). This means that the species feed quite frequently and the prey obtained from the food samples accounts for only about one-third of their daily consumption. Various sediment particles (e.g., sand grains) frequently observed in the faecal pellets were also reported by Hofrichter (1993). However, such particles were probably consumed incidentally with the prey and play no role in digestion to grind the food (as in birds, for example; [63]), as the digested prey was not crushed.

Observations in the aquarium show that all studied clingfish species rely mainly on vision to hunt, which is characteristic of many fish that sit in wait for prey [64,65]. Due to the wide range of prey taxa found in the diet of all three clingfishes, they could be considered carnivorous opportunists. This is consistent with the studies of other authors [23,29,66,67] and has also been observed in other clingfish species [68]. Indeed, highly opportunistic foraging for a wide range of small prey is a common characteristic of many cryptobenthic fish species [10,69].

In all three clingfish species, crustaceans were the most important prey group, which is consistent with many published studies [11,15,23,66,67,70,71,72]. The importance of crustaceans, especially amphipods, copepods, and decapods, in the diet of opportunistic cryptobenthic fish was also emphasised by Brandl et al. [69]. In all three clingfish species, harpacticoid copepods were the most abundant prey group, which has been observed in many fish species that feed on meiofaunal organisms (benthic organisms that pass through a 0.5 mm sieve and are retained on a 45 µm sieve; [73]). A high proportion of relatively small harpacticoid copepods in the diet could be a consequence of the fact that they have a 35% higher caloric value than amphipods and the proportion of successful prey capture is much higher for harpacticoid copepods than for amphipods [74]. However, it is important to consider which species of copepods and amphipods were used in the study. The high caloric content and low catch costs are probably the main reasons for the large proportion of copepods in the diet of clingfish, although their jaw anatomy allows clingfishes to eat much larger prey. The jaw shape studied by Hofrichter [11] seems to be closely related to the choice of food. The jaw is longest in *L. lepadogaster* and least elongated in *A. incognitus*. Since clingfish cannot generate suction pressure, this jaw shape and the ability to open the jaw up to an angle of 60° [11,15] allow *L. lepadogaster* to grasp large prey such as decapod crustaceans. This is also reflected in their relatively high trophic position in relation to their small body size, which is characteristic of cryptobenthic fish [12]. Furthermore, the teeth of clingfish are not designed to crush their prey but only to hold it [11,15]. Consequently, the prey is eaten whole, and due to the rapid digestion, it is often relatively undigested and hard parts remain uncrushed. This is probably the reason why the faecal pellets contain a relatively thick peritrophic membrane that surrounds the prey remains and protects the intestinal walls from damage that could be caused by sharp prey remains [75]. Since the peritrophic membrane is formed in the midgut (D.T. personal observation), this suggests that all digestion takes place there.

The importance of crustaceans as prey for the clingfish is also reflected in the red courtship colouration, which is reflected in *L. lepadogaster* in bright red dorsal, anal, and caudal fins, in *L. candolii* in three to four oblique, bright red stripes on the gill cover and three to five oblique, bright red spots extending from the middle of the back to the middle of the basal part of the dorsal fin, and in *A. incognitus* the head of the male was intensely red [76]. Pigments or their precursors, especially carotenoids (red, orange, and yellow colours), cannot be synthesised but must be ingested with food, which means that colour expression depends on individual feeding success and physiological performance [77]. As carotenoids are limited in availability and one of their main sources is crustaceans (e.g., crabs; [78]), this could suggest that the red colouration of male clingfish is related to their feeding success, which depends on their ability to defend a territory (hiding place) that provides a sufficient number of crustaceans as prey. This is probably related to the habitat choice of mating males of *L. lepadogaster* and *L. candolii*, which are most often found near the intertidal border in the upper infralittoral [20], which is known to be one of the most productive areas [79]. This shows how important crustaceans are as a prey group for the studied clingfish species.

#### 4.1.1. *Lepadogaster lepadogaster*

The diet is consistent with the findings of other researchers. A high proportion of crustaceans, represented by copepods and amphipods, has been observed in the diet of many other tidewater fish species [23]. Amphipods and copepods have also been recognised as one of the most abundant prey groups in the diet of *Gobius paganellus* [66,80,81], a species frequently observed in the same habitat as *L. lepadogaster* (D.T. personal observation). The great importance of decapods and amphipods in the diet of *L. lepadogaster* was also observed by Wilson [82]. Thus, the high number and abundance of amphipods and decapods in the diet correspond to a high number and abundance of them among stones, indicating the opportunistic feeding habits of *L. lepadogaster*. Despite the high abundance of copepods, their importance (IRI) in the diet was rather low compared to decapods and amphipods, which is due to their negligible biomass. In fact, the average weight of decapods in the diet of *L. lepadogaster* was 1026 times greater than the average weight of copepods. This is consistent with the observations of King [29], who noted that copepods, despite their high abundance, represent only a small total volume of prey, while conversely, decapods, despite their low abundance, represent a large total volume of prey. However, the weights of prey in this study should be interpreted with caution as they were calculated using correlation curves (e.g., Decapoda) or average weights (e.g., Copepoda), so their weight may slightly differ from the actual values.

#### 4.1.2. *Lepadogaster candolii*

Despite their negligible biomass, copepods are the most important prey in terms of IRI due to their high proportion in the diet of *L. candolii*, followed by decapods, isopods, and amphipods. The importance of copepods and amphipods in the diet has also been observed by other authors [23,71,72]. The high proportion of decapods and amphipods in the diet is consistent with their high abundance and frequency of occurrence under the rocks inhabited by *L. candolii*. However, despite the high number of polychaets and ophiuroids under rocks, they were not as abundant in the diet of *L. candolii*. The reason for this could be that polychaets hide in the sediment or, in the case of serpulid polychaets, in tubes, making them less accessible to clingfishes, while ophiuroids do not seem to be a preferred prey, as they were rarely ingested.

The presence of parasitic copepods (family Caligidae), parasitic larvae of Gnathiidae, and fish scales in the diet suggests that *L. candolii* also feeds on fish parasites, which is consistent with the observations of Mazé [67]. These results support the finding that *L. candolii* is an occasional cleaner fish whose cleaning activity in the Mediterranean was first observed by Weitzmann and Mercader [83]. Among the Mediterranean clingfish species, cleaning behaviour was also observed in *Diplecogaster bimaculata* [84]. In a few cases, parasitic larvae of Gnathiidae were also found in the diet of *L. lepadogaster*. In addition, they were observed as parasites on specimens of *L. lepadogaster* and *L. candolii*, while parasitic copepods were also observed in the latter species (D.T. personal observation). Therefore, it is possible that there is an intraspecific cleaning behaviour in clingfish.

#### 4.1.3. *Apletodon incognitus*

This species has only recently been described [11,21] and this is the first detailed insight into the diet of this species. It is a small species, mostly associated with *P. nobilis* shells [20], where it feeds mainly on small crustaceans, of which harpacticoid copepods are by far the most important. Harpacticoid copepods have also been recognised as the most important food for the similar small stenotopic clingfish *Opeatogenys gracilis* (Canestrini, 1864), living on seagrass leaves [85], where we did not find any specimen of *A. incognitus*, but such observations have been reported by Hofrichter and Patzner [7].

### 4.2. Factors Affecting the Diet of Clingfishes

Various factors influence the diet of clingfish. Roughly, the factors can be divided into external environmental factors (e.g., food availability, season, habitat) and internal factors related to the fish itself (e.g., size of the fish, home range, behaviour). However, the factors are strongly intertwined and it is difficult to separate them completely. For example, the diet of clingfish can be influenced by the season, which in turn is related to the seasonal change in the fish behaviour (e.g., nesting) and the availability of prey (prey settlement and seasonal variability in abundance). In addition, the available food also depends on the depth and the habitat.

#### 4.2.1. Fish and Prey Size

Fish size is one of the most important factors in food selection [73]. A comparison of the average size of fish species shows that larger species have a larger food niche. This was observed in *L. lepadogaster* as the average largest of these three species with 47 taxa found in its diet, while 42 different prey taxa were recognised in the diet of the slightly smaller *L. candolii*, and only 23 different taxa were observed in *A. incognitus* as the smallest species. This was reflected also in the different ITD values among species (Table 3). These results are in line with previous findings of other authors [15,73,86], who emphasised that a larger body size allows fish to feed on a wider range of prey. However, the composition of the diet depends not only on the size of the fish but also on the energetic benefits derived from hunting certain prey [73]. The differences in prey composition and size with increasing fish size are related to the optimisation of energy intake for growth [87]. Therefore, larger predatory fish eat larger prey [73]. However, if they eat larger prey, the fish have to expend less for the same amount of food. Consequently, eating larger prey also leads to a lower average number of prey ingested, as observed in *L. lepadogaster*, which ingested the lowest average number of prey per fish (7.1) compared to *L. candolii* (14.8) and *A. incognitus* (16.6). In general, smaller specimens (smaller size classes) of *L. lepadogaster* and *L. candolii* feed mainly on small prey such as copepods, and with increasing size (larger size classes), the specimens move on to larger prey such as decapod crustaceans. This is also reflected in the TROPHs index, which increases with the size of the fish species and with the size of the specimens (size classes) within the species, which is consistent with the observations of Hayden et al. [12] in other cryptobenthic species. Small animals, such as copepods, are most likely to be eaten by young fish stages or those of small adult size [73]. Meiofauna (especially harpacticoid copepods) are an important prey group for fish in the size range from 30 to 60 mm, depending on jaw structure [73]. As has been observed, the size of these three clingfishes allows them to still feed efficiently on copepods, which enables them to meet their energy requirements in the absence of larger prey, especially during the breeding season. In the smaller *A. incognitus*, copepods were the most important prey even at the adult stage, and a switch to larger prey was not observed. This is probably due to the small size of the adults, which allows efficient utilisation of smaller prey, while it could be also connected to the nesting behaviour.

#### 4.2.2. Home Range and Behaviour

The limited distribution range of cryptobenthic fish species, which naturally restricts access to prey [10], could explain their opportunistic diet. Species with a smaller home range are therefore more likely to feed opportunistically on a wide range of prey than species with a large distribution range. This is confirmed by the ITD value, since the dietary diversity indicates the degree of opportunistic feeding, which was highest in *L. lepadogaster*. This species lives the most cryptically of all three species, has the smallest home range, and is highly territorial [18]. Based on the underside of the stone [20], *L. candolii* had a larger home range and also fed outside the shelter [88,89], which is reflected in a less opportunistic diet (ITD = 0.73). *A. incognitus* was the species with the least cryptobenthic lifestyle (most often found outside the shelter [20]), so it is considered to have the largest home range of all three species, which is reflected in the least opportunistic diet of all three species (ITD = 0.51). However, it is not clear which has a stronger effect on the composition of dietary diversity in these three species, the home range or the fish size, as both explain the results well.

The behaviour of the fish also proved to be an important factor influencing the composition of the fish diet. The aquarium observations showed that *L. lepadogaster* moved mainly inside the space below the stone, while *L. candolii* and *A. incognitus* were much more mobile and moved outside the shelter (e.g., on the tops of the stones). This feeding behaviour was also reflected in the composition of the diet, as the specimens of *L. candolii* and *A. incognitus* had more prey in their diet that normally occur on the top of stones on algae (e.g., Cumacea and Caprellidae) than *L. lepadogaster*.

Nesting behaviour, which is related to the time of year, also affects the diet of the fish. During the breeding season, the males take care of the nest, so their movements are limited to the nest [15]. Consequently, they do not move around in search of food but are mainly dependent on prey that comes or is carried into the immediate vicinity of the nest by the water current. The high proportion of copepods in the males’ diet could therefore be related to their territorial behaviour and nest guarding. The higher proportion of copepods in the diet of males was observed in all three species, although they were larger than the females and would be expected to feed more on larger prey such as decapods. The importance of copepods in the diet of *L. lepadogaster* males was observed especially during the mating season (spring), when copepods were the most significant prey, while outside the mating season, copepods were much less important. It should be noted that infralittoral harpacticoid copepods are most abundant in the Gulf of Trieste in summer [90], but there are no data on copepods from the mediolittoral. Furthermore, copepods were found to play an important role in the diet of nesting males of *A. incognitus*, which is probably related to their hiding mode, as larger individuals (which were mostly nesting males) usually hide in oyster shells attached to the shells of *P. nobilis* and therefore rely mainly on prey that happens to swim/come into the oyster shells (e.g., copepods). This is also reflected in the less diverse diet and lower TROPHs index of larger specimens (mostly nesting males) of *A. incognitus* compared to smaller ones. This type of diet is possible due to the extensive diurnal migration of harpacticoid copepods between sediment and grass blades [91,92], which thus enter empty oysters on *P. nobilis* in seagrass beds. In addition, *A. incognitus* nests in summer, when infralittoral harpacticoid copepods are most abundant in the Gulf of Trieste [90]. However, feeding the males with copepods may result in larger prey (e.g., decapods) being available to the females during the mating season, as these require more energy for egg production.

#### 4.2.3. Food Availability

##### Depth and Habitat

The differences in the diet of clingfish depend on their selectivity and on the availability of different prey. The availability of different prey depends on the bottom depth and the associated occurrence of different habitats. The differences in diet composition as a function of depth were most evident in *L. lepadogaster* and *L. candolii*. In both species, the largest individuals were observed to be most abundant just below the tide line [20]. This rocky upper infralittoral is also known as a habitat for brown algal forests of the genus *Cystoseira* [93], which is among the most productive habitats [94] and provides niches for various species of invertebrates such as molluscs and crustaceans [93,95,96], which are potential food for clingfish. However, the presence of large specimens of both species in this area is probably related to the availability of decapods, which were their main prey. Based on the identification characteristics of the prey [49], the decapods belonged to the genus *Pisidia*, more specifically to *P. bluteli* or *P. longimana*. These two species, together with *A. nitescens*, make up most of the decapods in the diet of *L. lepadogaster and L. candolii*. In addition, crabs of the genus *Pisidia* are also known to be important food for many other fish species [97]. *P. bluteli* and *P. longimana* are mainly found in the upper infralittoral near the tide line, where they hide under stones [46,98], while *A. nitescens* occurs on a mixture of sediment, phytal, and hard bottom [46], being most abundant on pebbles in the upper infralittoral [99]. This is consistent with our results on fauna sampling under stones, which show that *A. nitescens* is more abundant in the habitat of *L. candolii*, which is also reflected in the composition of the diet. In fact, *L. candolii* had a higher proportion of *A. nitescens* in its diet than *L. lepadogaster*. Thus, the high proportion of decapods in the diet of *L. lepadogaster* and *L. candolii* in the lower mediolittoral and upper infralittoral is consistent with the presence of decapods in this habitat, while the high proportion of copepods in the diet of both species away from the tide line is related to the presence of smaller specimens and alternative prey at these depths. In addition, the large number of copepods in the diet of *L. candolii* from deeper waters may be related to the fact that the sand deeper on the seabed replaces the pebbles. Consequently, the proportion of decapods that prefer pebbles as substrate in the diet of *L. candolii* decreases with depth, while the proportion of harpacticoid copepods in the diet that live in/on sand [91] increases with depth. These results suggest that the preferred prey determines the presence and abundance of clingfish species, as well as the specimens of a certain size. This is not surprising, as cryptobenthic fish appear to have a higher mass-specific metabolic rate than larger fish species due to their small body size [69]. Consequently, energy consumption in small fish is very high and they barely tolerate periods of starvation or reduced food intake [100]. Small fish are therefore dependent on constant feeding with high-quality food (e.g., crustaceans) [69], the exploitation of which is limited by the size of the prey that the fish can catch and ingest [101].

##### Season

Seasonal differences were found in the occurrence of certain prey in the diet of *L. lepadogaster*, whereas this factor was not tested in *L. candolii* and *A. incognitus*, due to the uneven distribution of the fish sample over the seasons. The summer peak of decapods in the diet of *L. lepadogaster* coincides with a high abundance of decapod larvae in summer in temperate latitudes [102]. No reproductive data are available for *P. bluteli* and *P. longimana*, whereas the closely related species *P. longicornis*, which lives in somewhat deeper waters, is known to have a summer larval colonisation. In addition, the highest mortality was observed in this species in summer [97]. The newly settled *Pisidia* are most numerous at the end of summer and the beginning of fall [97], which explains their high abundance and frequency in the diet of clingfish at this time. This is also confirmed by the results showing that specimens of *Pisidia* sp. were on average the smallest in the diet of *L. lepadogaster* in summer and especially in autumn. Since *L. lepadogaster* and *Pisidia* sp. are low-mobility species [15,97], this probably means that when the fish eat most of the *Pisidia* sp. in their hiding places, the abundance and frequency of *Pisidia* sp. in the diet consequently decreases, which was observed in winter and spring. In times of shortages of suitable decapods, *L. lepadogaster* increasingly feeds on other prey groups (e.g., snails and copepods). Such a shift in diet from decapods and isopods in the warmer season to molluscs in the colder season was also observed by Compaire et al. [103]. Furthermore, this is consistent with Zander and Hagemann [104], who reported that fish feed on alternative prey in spring when there is a shortage of benthic crustaceans of suitable size. However, the decline of decapods in the diet of *L. lepadogaster* may also be due to decapods reaching a size at which they are difficult or impossible for fish to eat; however, this is less likely for *P. bluteli* and *P. longimana* with a relatively small size at which they can still be eaten by adult *L. lepadogaster* specimens.

Fish eggs’ presence in the diet is also related to the season and nesting behaviour. The feeding on fish eggs by *L. candolii* and *L. lepadogaster* was previously observed by Almada et al. [88] and Hofrichter [11]. Fish eggs in the diet could be the result of predation on other fish nests, the removal of the remains of their own hatched eggs, or, most likely, cannibalism. Cannibalism can occur when individuals prey on the nests of other males or when nesting males prey on their own eggs due to the shortage of food during the nesting season (small amount of potential prey in the immediate vicinity of the nest). Guarding males therefore sometimes eat some or all of their own eggs to keep themselves alive during the nesting season [105]. This cannibalism was particularly observed in *A. incognitus*, as fish eggs were usually found in the faecal pellets of the nesting males. Such consumption of eggs from their own spawn was also observed in guarding males of *Gobius auratus* Risso, 1810 [104]. Another interesting prey item ingested by nesting males of *A. incognitus* was serpulid polychaets, which were frequently observed in occupied oyster shells. This could indicate that serpulid polychaets are also an alternative prey during the mating season when the males take care of the nest and their movements are restricted to the oyster shell. Fish feeding with polychaets at a time when there is a lack of more suitable food (in spring) has been observed previously by Zander and Hagemann [104].

## 5. Conclusions

The faecal pellet-based method proved to be efficient in its application to clingfish, as all specimens survived the method and faecal pellets were obtained from 96.9% of all specimens examined.

Based on their diet, all three clingfishes *Lepadogaster lepadogaster*, *L. candolii*, and *Apletodon incognitus* can be considered carnivorous opportunists that feed mainly on crustaceans, with amphipods, copepods, and decapods being the most important taxa. The greatest overlap in diet occurred between *L. candolii* and *L. lepadogaster*, and the least between *A. incognitus* and *L. lepadogaster*.

In all three species, differences were found in the diet of specimens of different size classes, while in *L. lepadogaster* and *L. candolii*, differences were also observed in the diet of specimens from different bottom depths. Differences in diet between the sexes were only observed in *A. incognitus*, while seasonal differences in diet were confirmed in *L. lepadogaster*. Diet composition depends on the species of clingfish, the size of the specimens and the size of the prey they can eat, the behaviour of the fish and the associated home range of the specimens, and also on the availability of food, which depends on habitat, depth, and season.

The presence of certain crustacean taxa in the environment determines the occurrence of clingfish of different species and sizes.

The study of fauna as potential prey and observation in the aquarium have both proven to be very useful methods and considerably complement the results of diet composition in order to obtain a comprehensive understanding of the dietary habits of the species.

## Figures and Tables

**Table 1:** Size and age classes of three clingfish species as proposed by Hofrichter [11].

Size Class	Age Class (years)	*L. candolii*	*L. lepadogaster*	*A. incognitus*
1	0+	<25 mm	<22 mm	<16 mm
2	1+	26–39 mm	23–37 mm	17–24 mm
3	2+	40–51 mm	38–50 mm	25–34 mm
4	3+	52–70 mm	51–63 mm	>35 mm
5	4+	>71 mm	>64 mm	-

**Table 2:** Diet attributes of three clingfish species obtained with a non-destructive method.

	*L. lepadogaster*	*L. candolii*	*A. incognitus*
Proportion of defecated specimens (%)	96.9	96.8	97.4
Number of defecated specimens	188	120	37
Number of faecal pellets	295	161	56
Average number of faecal pellets per fish	1.6	1.3	1.5
Number of prey items per species	1334	1779	749
Average number of prey items per fish	7.1	14.8	16.6
Maximum number of prey items per fish	39	143	131
Minimum number of prey items per fish	1	1	1
Average number of prey items per faecal pellet	4.5	11.0	12.7
Maximum number of prey items per faecal pellets	39	143	70
Minimum number of prey items per faecal pellets	1	1	1

**Table 3:** ITD and TROPHs indexes for three clingfish species. Some classes were grouped together due to the small sample size.

Size Class	ITD			TROPHs		
	*L. lepadogaster*	*L. candolii*	*A. incognitus*	*L. lepadogaster*	*L. candolii*	*A. incognitus*
1	-	0.63	-	-	3.03	-
2	-	0.69	0.78 *	-	3.14	3.26 *
3	0.75 *	0.76	0.31	3.18 *	3.19	3.05
4	0.84	0.81 **	0.68 **	3.21	3.22 **	2.98 **
5	0.82	-	-	3.35	-	-
Per species	0.83	0.73	0.51	3.18 ± 0.44	3.12 ± 0.36	3.13 ± 0.26

* The group also contains specimens of a smaller class; ** the group also contains specimens of a larger class.

**Table 4:** Bray–Curtis dissimilarity index of three clingfish species.

	*L. candolii*	*A. incognitus*
*A. incognitus*	0.445	-
*L. lepadogaster*	0.391	0.458

**Table 5:** Occurrence of different prey groups under stones in the habitat of *L. lepadogaster* and *L. candolii*.

		*L. lepadogaster*		*L. candolii*	
Higher taxon	Lower taxon	N(%)	F(%)	N(%)	F(%)
Mollusca	Gastropoda	3.7	55.0	0.4	30
	Bivalvia	1.4	30.0	2.0	70
Crustacea	Decapoda	11.5	75.0	16.5	100
	Amphipoda	63.1	100.0	9.6	100
	Anisopoda	-	-	0.2	20
	Isopoda	3.8	50.0	1.3	60
	Mysida	2.6	65.0	0.2	10
Pantopoda	Pycnogonida	0.1	5.0	0.1	10
Annelida	Polychaeta	10.9	90.0	56.5	100
Echinodermata	Ophiuroida	2.9	70.0	13.0	100

## Data Availability

Data are contained within the article and Supplementary Materials.

## Supplementary Materials

The following supporting information can be downloaded at: https://www.mdpi.com/article/10.3390/ani14192835/s1, Table S1: Diet composition of *Lepadogaster lepadogaster*, *L. candolii*, and *Apletodon incognitus*.
